# Reliability of the tunisian pediatric gait, arms, legs, and spine: toward a valid screening tool for tunisian children with musculoskeletal conditions

**DOI:** 10.1186/s12969-023-00905-9

**Published:** 2023-11-02

**Authors:** Lassoued Ferjani Hanene, Narjess Amdouni, Rabhi Emna, Sahli Hela, Ben Abdelgheni Kaouther, Ben Nessib Dorra, Kaffel Dhia, Maatallah Kaouther, Hamdi Wafa

**Affiliations:** 1 Rheumatology department, Kassab orthopedics institute, Ksar Saïd, Tunisia; 2 Faculty of Medicine of Tunis, University Tunis el Manar, Ksar Saïd, Tunisia; 3 Research Unit UR17SP04, Ksar Said, Tunis, 2010 Tunisia; 4 Pediatric out clinics, Tbourba Hospital, Ksar Said, Tunis, Tunisia; 5 Rheumatology department, Rabta Hospital, Tunis, Tunisia; 6 Rheumatology department, Mongi Slim Hospital, Tunis, Tunisia

**Keywords:** pGALS, Physical examination, Screening, Musculoskeletal, Child, Tunisia

## Abstract

**Background:**

Pediatric musculoskeletal disorders account for 10% of first-line consultations in Tunisia. Referral delay and deficiencies in musculoskeletal screening raise a challenge to the early diagnosis and management of rheumatic conditions in children. The pGALS (Pediatric Gait Arms Legs Spine) was developed and translated into many languages to overcome these deficiencies. Our study aimed to adapt and validate pGALS to the Tunisian dialect for school-age children.

**Methods:**

Using the Delphi method, we carried out a cross-cultural adaptation of the pGALS to the Tunisian dialect. This consensual version was validated in a cross-sectional study, in two pediatric centers.

**Results:**

Ninety-two patients were enrolled, 43 females (46.7%) and 49 males (53.3%), mean age was 9.4 ± 2.6 years. The mean test duration was 3.4 ± 2.3 min, and the acceptability and comprehension of the test were good. Six patients had a musculoskeletal complaint, 19 had positive pGALS, and 14 were diagnosed with musculoskeletal disease. The internal consistency score (Cronbach’s α) was 0.852. The sensitivity of the test was 92.8%, the specificity was 92.3%, the positive likelihood ratio was 2.16, and the negative likelihood ratio was 0.01.

**Conclusion:**

The pGALS test adapted to the Tunisian dialect is a relevant, quick, and valid tool for screening musculoskeletal abnormalities in school-age children.

**Supplementary Information:**

The online version contains supplementary material available at 10.1186/s12969-023-00905-9.

## Background

In Tunisia, 10.4% of children visit primary health care for bone pain, arthralgia, or arthritis [1]. Musculoskeletal (MSK) conditions seem to be the fourth reason for pediatric consultation with less than a 4% referral rate to the specialist. However, access delay remains common, and for many children results in a prolonged period of untreated active disease which poorly impact the prognosis. The paucity of pediatric rheumatologists in Tunisia contributes to the referral delay. Such in many African countries, pediatric MSK clinical skills, and training in this field at African universities are deemed inadequate [2]. This raises a challenge to the early diagnosis and management of rheumatic conditions in children. To address this gap, many screening tools were developed worldwide [3, 4].

The pediatric Gait, Arms, Legs, and Spine (pGALS) was the most known test [5–7]. It was developed in 2006 by Helen Foster and her colleagues as a practical and acceptable method to differentiate between normal and abnormal MSK conditions in school-aged children [8, 9]. It has been shown to have good sensitivity and specificity for detecting MSK conditions.

However, the use of the original pGALS tool is limited in Tunisia due to the language barrier. Tunisian children primarily speak Tunisian and French, with a limited understanding of English. Therefore, to make the pGALS tool more accessible and applicable in daily practice, it is necessary to translate and validate it in the Tunisian dialect.

By adapting the tool to the Tunisian dialect, it will become more suitable for use in the local context, enabling primary healthcare professionals to effectively screen for MSK conditions in children and refer those with suspected conditions to the appropriate reference center in a prompt manner. Having a locally adapted screening test will improve certainly the early diagnosis and management of rheumatic conditions in Tunisian children, even in the absence of an adequate number of pediatric rheumatologists.

Therefore, our study aimed to perform a transcultural translation and validation of the pGALS screening tool in the Tunisian dialect.

## Methods

### Study design

Our study involved two steps: The first step was a transcultural translation of the pGALS into the Tunisian dialect. The second was the validation of the translated version through a cross-sectional multicenter study.

#### First step

We have adopted the original English version of the pGALS as starting point. After the author’s permission for the original version (H. Foster), translation into the Tunisian dialect was started using the following process:

-*Translation*: Two native bilingual speakers were required to translate the pGALS, they were informed by the study aim. The translation process was made according to the guidelines for the process of trans-cultural adaptation of self-report measures [10]. The two independent versions obtained were merged into the preliminary version after a deep discussion between the translators and the study supervisor (HL).
- *Delphi approach*: We choose to validate the Tunisian version using a Delphi method [11]. This approach was adopted in the consensus elaboration and recommendation building [12]. The expert panel was composed of two professors of adult rheumatologists (HS and KBA), two pediatric rheumatologists (WH and HL), and a general pediatrician (NA). All the group expert was informed of the study target and invited by email.

The items of the preliminary version of the Tunisian pGALS were mentioned in an online survey (Google Forms), and sent to the expert members to assess the translation of each item on a “Likert scale” ranging from 1 to 9.

The experts’ responses were collected and analyzed by the study supervisor: a median of responses between 1 and 3 defined an expert disagreement, between 4 and 6 defined an equivocal opinion and a median between 7 and 9 defined agreement. Consensus is reached for each item if at least 70% of the experts agree.

Three rounds of the Delphi were held. The results of the first vote were communicated to the experts by email and during the online meeting. This feedback concerned the median of responses, the spread of opinions about this median, and the suggestions put forward.

During the meeting, all the items were repeated one by one, to check the content validity of the Tunisian version of the pGLAS.

The items that did not reach the expert’s agreement were reformulated in light of the panel discussions and were the subject of the second round of Delphi. Electronic voting and the second online meeting led to a consensus agreement for all pGALS items. As a result, version 2 was obtained. The final version was sent by mail for expert members to approve the final result.

-*Back-translation*: The final version has been back-translated from the dialect Tunisian into English by an English teacher to check that there is no distortion compared with the original version. At the end of the back-translation process, the final version was deemed valid linguistically.
*- Pre-test*: The final version of the pGALS was tested on ten patients from the pediatric consultation to assess the test acceptability and comprehension.

#### Second step

We undertook a cross-sectional study in two centers: the general pediatrics output clinics and a tertiary pediatric rheumatology center. The study has been extended by three months: from 01/07/2022 to 31/10/2022.

### Study population

The patients in this study were school-age children: between 5 and 16 years old of age. The patients with psychomotor retardation, life-threatening emergencies, and physical trauma were excluded.

The final version was used on the children consulting the general pediatrics output clinics and a tertiary pediatric rheumatology center for any complaints (MSK or no MSK manifestations).

### pGALS assessment

To range an objective record for the test, the pediatrician, received the online training on the children examination through the PMM website (https://www.pmmonline.org/doctor/clinical-assessment/normal-variants-in-musculoskeletal-development/).

After the parent and guardant oral explanation of the study target, the pediatrician demonstrated the pGALS maneuvers with a “copy me” approach. The answer to three pGALS questions was obtained. The duration of the test was assessed by the manual stopwatch. The pGALS was considered positive if at least one question or maneuver was positive. The acceptability and comprehensibility of the test by the children and their parents or guardians were also evaluated using visual analog scores (0–10).

### Statistical analysis

The sample size was calculated using the following formula: n = Z² x p (1-p)/ m². For the descriptive statistical study of socio-demographic characteristics, the variables were described using means and standard deviations. The variables were described using numbers and percentages. As a screening tool, we studied the sensitivity, specificity, ratio of positive likelihood ratio, and negative likelihood ratio (weighted by the prevalence) with VassarStats: A website for statistical calculation.

We used SPSS software (version 23). The significance level was: p < 0.05. We studied the associations between the pGALS test and the MSK disease, using Pearson’s Chi-square test or Fisher’s exact test.

The reliability of the instrument was assessed by measuring the internal consistency via Cronbach’s alpha coefficient. The sampling adequacy was evaluated using the.

Kaiser-Meyer-Olkin (KMO) test, where values > 0.7 are considered good. Bartlett’s sphericity test was used to examine the null hypothesis that the variables were not correlated in the population (p < 0.05). External validity was not applicable in our study because of the lack of a gold standard tool.

Construct validity was estimated by factor analysis of different items.

For the exploratory factor analysis, we used the Maximum likelihood and Varimax orthogonal rotation with three factors solution, the eigenvalue was calculated using the scree plot method [13]. Items with the highest loading (> 0.40) were used to interpret factors.

### Ethical concern

The study was approved by the local ethical committee. Verbal consent was approved by parents or guardians after explaining the aims of the study.

## Results

### Patients characteristics

The Tunisian pGALS was proposed to 95 eligible patients; three patients did not consent. In the end, 92 patients participated in the study: 43 girls (46.7%) and 49 boys (53.3%). Four patients had chronic diseases (three diabetics and one patient with sickle cell disease). The socio-demographic characteristics of the patients are detailed in Table 1.

**Table 1:** Sociodemographic data of the study population

Variables	
Age (years)	9.4 ± 2.6
Birth weight (gr)	3155 ± 516
Obesity; n(%)	3 (3.2)
Growth delay; (%)	11(11.9)
Normal term at birth ; n(%)	88 (95.7)
Pre-maturity ; n(%)	3 (3.3)
Post-maturity ; n(%)	1(1.1)
Family history of MSK; n(%)	5 (5.4)
Age of walking ; mean ± SD (months)	15.9 ± 3.4

*MSK: musculoskeletal, gr: grams; SD: standard deviation*

### Feasibility and acceptability of the tunisian pGALS 

The pGALS spent the meantime at 3.4 ± 2.3 min. The means of children’s acceptability and comprehensibility for pGALS were 9.1 ± 1.2 and 9.3 ± 0.86; respectively. Similarly, parent’s acceptability and comprehensibility were at 9.6 ± 0.7 and 9.6 ± 0.6; respectively.

### Evaluation of the tunisian pGALS 

During the study, six patients complained of MSK manifestation: rachialgia (n = 2), polyarthritis (n = 1), hip and knee pain (n = 2), and mid-thigh pain (n = 1). Other patients presented for various ambulatory pediatric reasons as follows: fever, rash, abdominal pain, diarrhea….

The pGALS was positive in 19 patients (20.6%). The presenting manifestations in patients with abnormal pGALS are summarized in Table 2.

**Table 2:** Patients with abnormal Pediatric Gait, Arms, legs, and Spine assessment

Presenting complaint	Component altered on the pGALS	Final diagnostic
Polyarthralgia	Gait, TMJ, Arms	JIA
Diabetes Follow-up	Trigger fingers	Diabetic
Diabetes Follow-up + fracture history	Trigger fingers	Diabetic
Lumbar pain	Spine deviation	Scoliosis
Cough	Spine deviation	Scoliosis
urinary burning	Foot abnormality	flat foot
Asthma	Knee movement limitation	No MSK
Asthma	Pectus excavatum	Rickets
Lumbar pain	Foot abnormality	Flat foot
Hip pain/sickle cell disease	Painful hip	Vaso-occlusive crisis
Knee pain	Positive knee test	Anterior cruciate ligament hypersignal on MRI
Hip pain/asthma/ Enuresis	Posture asymmetry dorsal Hyperkyphosis	Scoliosis
Asthma	Elbow hyperextension	Hypermobility
Itchy skin	cross-fluctuation on the knee	No MSK
Growth delay	toes abnormalities	Syndactilya
Enuresis	Sacral dimple	No MSK
Fever/diarrhea	Gait abnormalities	No MSK
Skin rash	Spine deviation	Scoliosis
Headache	Painful knee	Hypermobility

*TMJ: temporomandibular joint; JIA: juvenile idiopathic arthritis; MSK: musculoskeletal; MRI: magnetic resonance imaging; pGALS: pediatric Gait, Arms, legs, and Spine*

The final diagnosis showed that 14 of the 19 patients with positive pGALS had MSK disease. Positive pGALS was associated with MSK disease (p < 0.001). This statistical association was found for both questions and maneuvers.

The sensitivity of the test was 92.8%, the specificity was 92.3%, the positive likelihood ratio was 2.16, and the negative likelihood ratio was 0.01. Question Q1 and maneuver M1 were positive in 8 and 11 cases; respectively, with significant association with the MSK disease (p < 0.001). The sensitivity and the specificity of Q1, M1, questions and maneuvers are detailed in Table 3.

**Table 3:** Sensitivity and Specificity of the Tunisian Pediatric Gait, Arms, legs, and Spine

	Q1	M1	Questions	Maneuvers	Total pGALS
Sensitivity (%)	57.1	64.2	50	92.8	92.8
Specificity (%)	100	97.4	100	92.3	92.3

*Q: question, M: Maneuvers; pGALS: pediatric Gait, Arms, legs, and Spine*

### Reliability of the tunisian pGALS 

The internal consistency study found overall Cronbach’s Alpha coefficient (of the 19 items) to be 0.852, questions to 0.613, and maneuver to 0.807. The measurement of KMO was 0.5. Barlett’s sphericity test showed significance (p < 0.001).

We were therefore able to reject the null hypothesis that the correlation matrix was an identity matrix and that there was no relationship between the items (**supplementary material**).

Analysis of the principal components of the items revealed 3 main factors: one first factor F1 grouping: Q2, Q3, M2, M3, M7, M8, M9, M10, M12, M14, M15; one 2nd factor F2 bringing together: Q1 and M13. The 3rd factor F3 regrouped M16 and M1.

## Discussion

The study aimed to validate the Tunisian version of the pGALS as a screening tool for MSK conditions in pediatric consultations in Tunisia. The process involved two stages: linguistic and metric validation. The translation encountered difficulties with the terms “patellar test” and “cross fluctuation test” in the Tunisian dialect. To ensure the understanding of the tool by both doctors and patients, it was important to use a dialect that school-age patients could conceive while maintaining a medical discourse for doctors who commonly used the French language.

The final Tunisian version of the pGALS spent an average test duration of 3.4 min. This additional time showed not perturbed the daily practice of the children’s exam even in an emergency context. Previous literature reported varying test durations, ranging from 2.2 to 5 min [14–17]. Studies that utilized an adapted version tended to have shorter test times compared to those that did not adopt the test due to language barriers [14, 18]. Additionally, the duration varied depending on the specialty, being shorter in emergency settings compared to physical medicine and rehabilitation, and it also varied based on the number of abnormalities detected [3, 14, 19, 20].

Similar to previous studies, the Tunisian version of the pGALS was found to be well-accepted by children and their parents. The internal criteria of the Tunisian version showed the sensitivity of the test was 92.8%, the specificity was 92.3%, the positive likelihood ratio was 2.16, and the negative likelihood ratio was 0.01. These findings were consistence with the Spanish, Italian, and Mexican studies which found high sensitivity and specificity ranging between 93 and 100% [15, 18].

Abnormalities in Q1 and M1 were frequently observed in the Tunisian version. Positive Q1 was associated with MSK conditions (p < 0.001) and seems to be a discriminant question in the pGALS. M1 yielded positive results in 11 out of 19 patients, emphasizing the importance of overall assessment of the child, both statically and while walking. However, some items of the pGALS were not recorded, as observed in a Colombian study [21].

Regarding the test reliability, the KMO measure was poor, indicating the independence of the pGALS items. Bartlett’s sphericity test result was significant, rejecting the null hypothesis. Cronbach’s Alpha values ranged between 0.6 and 0.8 for the pGALS as a whole and its components (questions and maneuvers), indicating that the translated questionnaire had an acceptable internal.

consistency.

Principal component analysis was employed to group items describing similar dimensions. This analysis identified three clusters: F1 for peripheral joint examination and functional signs, F2 for temporomandibular joint involvement, and F3 for evaluating the axial skeleton and postural syndrome. This statistical grouping demonstrated that the pGALS explores the entire musculoskeletal system in children. No previous study has explored this dimension using factor analysis.

## Conclusion

Our study successfully obtained a version of the pGALS in the Tunisian dialect, adapted for the local population. This version proved to be time-efficient, acceptable, and relevant in general pediatric consultations. While the pGALS is a simple and cost-effective tool, the examiner must consider the physiological variants of a child’s skeleton to avoid unnecessary concerns for parents in case of a test abnormality and to avoid burdening specialist consultations.

### Electronic supplementary material

Below is the link to the electronic supplementary material.

Supplementary Material 1

Supplementary Material 2

## Data Availability

The datasets used and/or analysed during the current study are available from the corresponding author on reasonable request.

## Abbreviations

F: Factor
KMO: Kaiser-Meyer-Olkin
MSK: musculoskeletal
pGALS: pediatric Gait, Arms, Legs,and Spine
M: maneuver
Q: question

## References

[1] Ajmi TN, Amor H, Bougmiza I, Gataa R, Mtiraoui A (2010). [Diagnoses in children seen in general practice in the region of Sousse (Tunisie)]. Sante Publique.

[2] Migowa AN, Hadef D, Hamdi W, Mwizerwa O, Ngandeu M, Taha Y et al. Pediatric rheumatology in Africa: thriving amidst challenges. Pediatr Rheumatol Online J. 7 mai 2021;19(1):69. 10.1186/s12969-021-00557-7 PMC8103667 33962643

[3] Tomaru Y, Kamada H, Tsukagoshi Y, Nakagawa S, Tanaka K, Takeuchi R (2019). Screening for musculoskeletal problems in children using a questionnaire. J Orthop Sci janv.

[4] Yamaguchi N, Chosa E, Yamamoto K, Kawahara K, Hamada H, Taniguchi N (2016). Screening for musculoskeletal problems in japanese schoolchildren: a cross-sectional study nested in a cohort. Public Health oct.

[5] Foster HE, Eltringham MS, Kay LJ, Friswell M, Abinun M, Myers A (2007). Delay in access to appropriate care for children presenting with musculoskeletal symptoms and ultimately diagnosed with juvenile idiopathic arthritis. Arthritis Rheum 15 août.

[6] Legault EP, Cantin V, Descarreaux M (2014). Assessment of musculoskeletal symptoms and their impacts in the adolescent population: adaptation and validation of a questionnaire. BMC Pediatr 3 Juill.

[7] Jinguji TM (2013). Musculoskeletal screening in children. Pediatr Ann nov.

[8] Foster HE, Jandial S (2013). pGALS - paediatric gait arms legs and spine: a simple examination of the musculoskeletal system. Pediatr Rheumatol Online J 12 Nov.

[9] Foster H, Kay L, May C, Rapley T (2011). Pediatric regional examination of the musculoskeletal system: a practice- and consensus-based approach. Arthritis Care Res.

[10] Beaton DE, Bombardier C, Guillemin F, Ferraz MB (2000). Guidelines for the process of cross-cultural adaptation of self-report measures. Spine (Phila Pa 1976) 15 déc.

[11] McPherson S, Reese C, Wendler MC (2018). Methodology update: Delphi Studies. Nurs Res.

[12] Hasson F, Keeney S, McKenna H (2000). Research guidelines for the Delphi survey technique. J Adv Nurs oct.

[13] Strickland OL (2003). Using factor analysis for validity assessment: practical considerations. J Nurs Meas.

[14] Palermi S, Annarumma G, Spinelli A, Massa B, Serio A, Vecchiato M (2022). Acceptability and practicality of a Quick Musculoskeletal Examination into Sports Medicine Pre-Participation evaluation. Pediatr Rep 3 mai.

[15] Abernethy K, Jandial S, Hill L, Sánchez ES, Foster H (2014). Acceptability and practicality of a spanish translation of paediatric gait arms legs and spine (pGALS) in peruvian children. Pediatr Rheumatol Online J 20 Nov.

[16] Smith E, Molyneux E, Heikens GT, Foster H (2012). Acceptability and practicality of pGALS in screening for rheumatic disease in malawian children. Clin Rheumatol avr.

[17] Batu ED, Keniş Coşkun Ö, Sönmez HE, Karali D, Arslanoğlu Aydin E, Bilginer Y (2017). Acceptability and practicality of the turkish translation of Pediatric Gait arms legs and spine in turkish children. J Clin Rheumatol déc.

[18] Moreno-Torres LA, Hernández-Garduño AG, Arellano-Valdés CA, Salinas-Rodríguez A, Rubio-Perez N, Peláez-Ballestas I (2016). Cross-cultural validation of the paediatric gait, arms, legs, spine (pGALS) tool for the screening of musculoskeletal disorders in mexican children. Rheumatol Int avr.

[19] Sukharomana M, Charuvanij S. The Thai Translation of the Pediatric Gait, Arms, Legs, Spine Tool is Useful for Pediatric Residents in Detecting Musculoskeletal Abnormalities in Children. J Clin Rheumatol. 31 mars 2020. 10.1097/RHU.0000000000001372 32251062

[20] Purusha A, Dhage D, Phansopkar PP, Arora P. SP. Orthopedic screening using pGALS assessment tools in children between 5 and 12 years. JMPAS. 30 janv 2022;11(1):4405–8.

[21] Cárdenas Silva AM, Rodríguez Perea LM, Avendaño Valdés K, Cuellar Zapata N, Valencia AM (2020). Gómez Mora M del P. usefulness of pGALS as a screening test in children and adolescents in Colombia. Cross-sectional study. Rev Colomb Reumatol 1 Juill.

