# Supplementary material for: Reliability of the tunisian pediatric gait, arms, legs, and spine: toward a valid screening tool for tunisian children with musculoskeletal conditions
Source: Pediatr Rheumatol Online J. 2023 Nov 2;21:133. doi: 10.1186/s12969-023-00905-9 (PMC10621183; doi:10.1186/s12969-023-00905-9)
Supplement: Supplementary file 1 — Supplementary Material 1 [file 12969_2023_905_MOESM1_ESM.docx]

| \|  \| \| \| Q1 \| Q2 \| Q3 \| M1 \| M2 \| M3 \| M 7 \| M 8 \| M 9 \| M 10 \| M12 \| M 13 \| M14 \| M15 \| M 16 \|  \|  \| \| --- \| --- \| --- \| --- \| --- \| --- \| --- \| --- \| --- \| --- \| --- \| --- \| --- \| --- \| --- \| --- \| --- \| --- \| --- \| --- \| \|  \| Q1 \| R \| 1,000 \| ,340^**^ \| ,483^**^ \| ,362^**^ \| ,340^**^ \| ,161 \| ,340^**^ \| ,378^**^ \| ,340^**^ \| ,340^**^ \| ,340^**^ \| ,340^**^ \| ,313^**^ \| ,340^**^ \| -,032 \|  \|  \| \| P \| . \| ,001 \| ,000 \| ,000 \| ,001 \| ,126 \| ,001 \| ,000 \| ,001 \| ,001 \| ,001 \| ,001 \| ,002 \| ,001 \| ,760 \|  \|  \| \| Q2 \| R \| ,340^**^ \| 1,000 \| ,703^**^ \| ,284^**^ \| 1,000^**^ \| ,571^**^ \| 1,000^**^ \| ,571^**^ \| 1,000^**^ \| 1,000^**^ \| 1,000^**^ \| -,011 \| ,492^**^ \| ,340^**^ \| -,032 \|  \|  \| \| P \| ,001 \| . \| ,000 \| ,006 \| . \| ,000 \| . \| ,000 \| . \| . \| . \| ,917 \| ,000 \| ,001 \| ,760 \|  \|  \| \| Q3 \| R \| ,340^**^ \| 1,000 \| ,703^**^ \| ,284^**^ \| 1,000^**^ \| ,392^**^ \| ,703^**^ \| ,392^**^ \| ,703^**^ \| ,703^**^ \| ,703^**^ \| -,016 \| ,334^**^ \| ,703^**^ \| -,016 \|  \|  \| \| P \| ,001 \| . \| ,000 \| ,006 \| . \| ,000 \| ,000 \| ,000 \| ,000 \| ,000 \| ,000 \| ,882 \| ,001 \| ,000 \| ,882 \|  \|  \| \| M1 \| R \| ,340^**^ \| 1,000 \| ,703^**^ \| ,284^**^ \| 1,000^**^ \| ,121 \| ,284^**^ \| ,121 \| ,284^**^ \| ,284^**^ \| ,284^**^ \| -,039 \| ,086 \| ,284^**^ \| ,284^**^ \|  \|  \| \| P \| ,001 \| . \| ,000 \| ,006 \| . \| ,251 \| ,006 \| ,251 \| ,006 \| ,006 \| ,006 \| ,715 \| ,417 \| ,006 \| ,006 \|  \|  \| \| M2 \| R \| ,340^**^ \| 1,000 \| ,703^**^ \| ,284^**^ \| 1,000^**^ \| ,571^**^ \| 1,000^**^ \| ,571^**^ \| 1,000^**^ \| 1,000^**^ \| 1,000^**^ \| -,011 \| ,492^**^ \| ,284^**^ \| ,284^**^ \|  \|  \| \| P \| ,001 \| . \| ,000 \| ,006 \| . \| ,000 \| . \| ,000 \| . \| . \| . \| ,917 \| ,000 \| ,006 \| ,006 \|  \|  \| \| M3 \| R \| ,161 \| ,571^**^ \| ,392^**^ \| ,121 \| ,571^**^ \| 1,000 \| ,571^**^ \| ,311^**^ \| ,571^**^ \| ,571^**^ \| ,571^**^ \| -,019 \| ,261^*^ \| ,284^**^ \| ,284^**^ \|  \|  \| \| P \| ,126 \| ,000 \| ,000 \| ,251 \| ,000 \| . \| ,000 \| ,003 \| ,000 \| ,000 \| ,000 \| ,856 \| ,012 \| ,006 \| ,006 \|  \|  \| \| M 7 \| R \| ,340^**^ \| 1,000^**^ \| ,703^**^ \| ,284^**^ \| 1,000^**^ \| ,571^**^ \| 1,000 \| ,571^**^ \| 1,000^**^ \| 1,000^**^ \| 1,000^**^ \| -,011 \| ,492^**^ \| 1,000^**^ \| -,011 \|  \|  \| \| P \| ,001 \| . \| ,000 \| ,006 \| . \| ,000 \| . \| ,000 \| . \| . \| . \| ,917 \| ,000 \| . \| ,917 \|  \|  \| \| M 8 \| R \| ,378^**^ \| ,571^**^ \| ,392^**^ \| ,121 \| ,571^**^ \| ,311^**^ \| ,571^**^ \| 1,000 \| ,571^**^ \| ,571^**^ \| ,571^**^ \| -,019 \| ,261^*^ \| ,571^**^ \| -,019 \|  \|  \| \| P \| ,000 \| ,000 \| ,000 \| ,251 \| ,000 \| ,003 \| ,000 \| . \| ,000 \| ,000 \| ,000 \| ,856 \| ,012 \| ,000 \| ,856 \|  \|  \| \| M9 \| R \| ,340^**^ \| 1,000^**^ \| ,703^**^ \| ,284^**^ \| 1,000^**^ \| ,571^**^ \| 1,000^**^ \| ,571^**^ \| 1,000 \| 1,000^**^ \| 1,000^**^ \| -,011 \| ,492^**^ \| ,571^**^ \| -,019 \|  \|  \| \| P \| ,001 \| . \| ,000 \| ,006 \| . \| ,000 \| . \| ,000 \| . \| . \| . \| ,917 \| ,000 \| ,000 \| ,856 \|  \|  \| \| M10 \| R \| ,340^**^ \| 1,000^**^ \| ,703^**^ \| ,284^**^ \| 1,000^**^ \| ,571^**^ \| 1,000^**^ \| ,571^**^ \| 1,000^**^ \| 1,000 \| 1,000^**^ \| -,011 \| ,492^**^ \| ,571^**^ \| -,019 \|  \|  \| \| P \| ,001 \| . \| ,000 \| ,006 \| . \| ,000 \| . \| ,000 \| . \| . \| . \| ,917 \| ,000 \| ,000 \| ,856 \|  \|  \| \| M 12 \| R \| ,340^**^ \| 1,000^**^ \| ,703^**^ \| ,284^**^ \| 1,000^**^ \| ,571^**^ \| 1,000^**^ \| ,571^**^ \| 1,000^**^ \| 1,000^**^ \| 1,000 \| -,011 \| ,492^**^ \| ,571^**^ \| -,019 \|  \|  \| \| P \| ,001 \| . \| ,000 \| ,006 \| . \| ,000 \| . \| ,000 \| . \| . \| . \| ,917 \| ,000 \| ,000 \| ,856 \|  \|  \| \| M13 \| R \| ,340^**^ \| -,011 \| -,016 \| -,039 \| -,011 \| -,019 \| -,011 \| -,019 \| -,011 \| -,011 \| -,011 \| 1,000 \| -,022 \| -,011 \| -,011 \|  \|  \| \| P \| ,001 \| ,917 \| ,882 \| ,715 \| ,917 \| ,856 \| ,917 \| ,856 \| ,917 \| ,917 \| ,917 \| . \| ,833 \| ,917 \| ,917 \|  \|  \| \| M 14 \| R \| ,313^**^ \| ,492^**^ \| ,334^**^ \| ,086 \| ,492^**^ \| ,261^*^ \| ,492^**^ \| ,261^*^ \| ,492^**^ \| ,492^**^ \| ,492^**^ \| -,022 \| 1,000 \| -,011 \| -,011 \|  \|  \| \| P \| ,002 \| ,000 \| ,001 \| ,417 \| ,000 \| ,012 \| ,000 \| ,012 \| ,000 \| ,000 \| ,000 \| ,833 \| . \| ,917 \| ,917 \|  \|  \| \| M 15 \| R \| ,340^**^ \| 1,000^**^ \| ,703^**^ \| ,284^**^ \| 1,000^**^ \| ,571^**^ \| 1,000^**^ \| ,571^**^ \| 1,000^**^ \| 1,000^**^ \| 1,000^**^ \| -,011 \| ,492^**^ \| -,011 \| -,011 \|  \|  \| \| P \| ,001 \| . \| ,000 \| ,006 \| . \| ,000 \| . \| ,000 \| . \| . \| . \| ,917 \| ,000 \| ,917 \| ,917 \|  \|  \| \| M16 \| R \| -,032 \| -,011 \| -,016 \| ,284^**^ \| -,011 \| -,019 \| -,011 \| -,019 \| -,011 \| -,011 \| -,011 \| -,011 \| -,022 \| -,011 \| 1,000 \|  \|  \| \| P \| ,760 \| ,917 \| ,882 \| ,006 \| ,917 \| ,856 \| ,917 \| ,856 \| ,917 \| ,917 \| ,917 \| ,917 \| ,833 \| ,917 \| . \|  \|  \| |
| --- | --- | --- | --- | --- | --- | --- | --- | --- | --- | --- | --- | --- | --- | --- | --- | --- | --- | --- | --- | --- | --- | --- | --- | --- | --- | --- | --- | --- | --- | --- | --- | --- | --- | --- | --- | --- | --- | --- | --- | --- | --- | --- | --- | --- | --- | --- | --- | --- | --- | --- | --- | --- | --- | --- | --- | --- | --- | --- | --- | --- | --- | --- | --- | --- | --- | --- | --- | --- | --- | --- | --- | --- | --- | --- | --- | --- | --- | --- | --- | --- | --- | --- | --- | --- | --- | --- | --- | --- | --- | --- | --- | --- | --- | --- | --- | --- | --- | --- | --- | --- | --- | --- | --- | --- | --- | --- | --- | --- | --- | --- | --- | --- | --- | --- | --- | --- | --- | --- | --- | --- | --- | --- | --- | --- | --- | --- | --- | --- | --- | --- | --- | --- | --- | --- | --- | --- | --- | --- | --- | --- | --- | --- | --- | --- | --- | --- | --- | --- | --- | --- | --- | --- | --- | --- | --- | --- | --- | --- | --- | --- | --- | --- | --- | --- | --- | --- | --- | --- | --- | --- | --- | --- | --- | --- | --- | --- | --- | --- | --- | --- | --- | --- | --- | --- | --- | --- | --- | --- | --- | --- | --- | --- | --- | --- | --- | --- | --- | --- | --- | --- | --- | --- | --- | --- | --- | --- | --- | --- | --- | --- | --- | --- | --- | --- | --- | --- | --- | --- | --- | --- | --- | --- | --- | --- | --- | --- | --- | --- | --- | --- | --- | --- | --- | --- | --- | --- | --- | --- | --- | --- | --- | --- | --- | --- | --- | --- | --- | --- | --- | --- | --- | --- | --- | --- | --- | --- | --- | --- | --- | --- | --- | --- | --- | --- | --- | --- | --- | --- | --- | --- | --- | --- | --- | --- | --- | --- | --- | --- | --- | --- | --- | --- | --- | --- | --- | --- | --- | --- | --- | --- | --- | --- | --- | --- | --- | --- | --- | --- | --- | --- | --- | --- | --- | --- | --- | --- | --- | --- | --- | --- | --- | --- | --- | --- | --- | --- | --- | --- | --- | --- | --- | --- | --- | --- | --- | --- | --- | --- | --- | --- | --- | --- | --- | --- | --- | --- | --- | --- | --- | --- | --- | --- | --- | --- | --- | --- | --- | --- | --- | --- | --- | --- | --- | --- | --- | --- | --- | --- | --- | --- | --- | --- | --- | --- | --- | --- | --- | --- | --- | --- | --- | --- | --- | --- | --- | --- | --- | --- | --- | --- | --- | --- | --- | --- | --- | --- | --- | --- | --- | --- | --- | --- | --- | --- | --- | --- | --- | --- | --- | --- | --- | --- | --- | --- | --- | --- | --- | --- | --- | --- | --- | --- | --- | --- | --- | --- | --- | --- | --- | --- | --- | --- | --- | --- | --- | --- | --- | --- | --- | --- | --- | --- | --- | --- | --- | --- | --- | --- | --- | --- | --- | --- | --- | --- | --- | --- | --- | --- | --- | --- | --- | --- | --- | --- | --- | --- | --- | --- | --- | --- | --- | --- | --- | --- | --- | --- | --- | --- | --- | --- | --- | --- | --- | --- | --- | --- | --- | --- | --- | --- | --- | --- | --- | --- | --- | --- | --- | --- | --- | --- | --- | --- | --- | --- | --- | --- | --- | --- | --- | --- | --- | --- | --- | --- | --- | --- | --- | --- | --- | --- | --- | --- | --- | --- | --- | --- | --- | --- | --- | --- | --- | --- | --- | --- | --- | --- | --- | --- | --- | --- | --- | --- | --- | --- | --- | --- | --- | --- | --- | --- | --- | --- | --- | --- | --- | --- | --- | --- | --- | --- | --- | --- | --- | --- | --- | --- | --- | --- | --- | --- | --- | --- | --- | --- | --- | --- | --- | --- | --- | --- | --- | --- | --- | --- | --- | --- |

M : maneuver ; Q : question ; r : correlation coeficient ; p : significance
