# Supplementary material for: Reliability of the tunisian pediatric gait, arms, legs, and spine: toward a valid screening tool for tunisian children with musculoskeletal conditions
Source: Pediatr Rheumatol Online J. 2023 Nov 2;21:133. doi: 10.1186/s12969-023-00905-9 (PMC10621183; doi:10.1186/s12969-023-00905-9)
Supplement: Supplementary file 2 — Supplementary Material 2 [file 12969_2023_905_MOESM2_ESM.docx]

cover letter

Dear Dr. Editor-in-Chief,

We wish to submit an **original article** entitled " **Reliability of the Tunisian Pediatric Gait, Arms, Legs, and Spine: Toward a valid screening tool for Tunisian Children with musculoskeletal conditions”** for consideration for publication in your esteemed journal. In this paper, we described the steps of the translation and the validation of the Tunisian version of the valid screening tool.

Cognizant of the fact that we have a different socio-cultural background in Tunisia, we proposed solutions adapted to our culture to screen children with musculoskeletal conditions and improve their management.

We think that our study is suitable for publication in the collection “Context-Specific Paediatric Rheumatology Care-Lessons from Africa”. Our aim was to highlight the challenges pediatric rheumatology patients in Tunisia and showcase the strategy to help overcome them through socio-cultural adaptation of best clinical practice.

- We confirm that the final manuscript has been seen and approved by all the authors.
- We have no conflicts of interest.
- We confirm that this work is original and has not been published elsewhere, nor is it currently under consideration for publication elsewhere
- we confirm that we have obtained the required ethical approvals

.
